# Something is up in the air: pneumothorax and pneumopericardium in a 29-week preterm infant

**DOI:** 10.1007/s10354-023-01021-9

**Published:** 2023-09-26

**Authors:** Sascha Meyer, Sarah Ruffing, Martina Geipel, Martin Poryo, Alexander Larsen, Sogand Nemat

**Affiliations:** 1 Department of General Pediatrics and Neonatology, University Hospital of Saarland, Building 9, 66421 Homburg, Germany; 2 Department of Pediatric Cardiology, University Hospital of Saarland, Homburg, Germany; 3 Department of Radiology, and Interventional Radiology, University Hospital of Saarland, Homburg, Germany

**Keywords:** Premature infant, Differential diagnosis, Respiratory distress syndrome, newborn, Neonatology, Echocardiography

## Abstract

This report highlights the early and unusual detection of a pneumopericardium by echocardiography prior to potential development of cardiocirculatory compromise. It is important to consider pneumopericardium into the differential diagnosis when difficulties arise in the visualization of the heart by conventional echocardiography. Pneumopericardium is associated with a high mortality rate and may be effectively treated by immediate insertion of a pericardial catheter.

**Video online:**

The online version of this article contains one video. The article and the video are available online (10.1007/s10354-023-01021-9). The video can be found in the article back matter as “Electronic Supplementary Material”.

## Case report

A 29 2/7-week-old premature neonate with a birth weight of 1400 g was born by spontaneous vaginal delivery because of maternal vaginal hemorrhage and premature contractions. Apgar scores at 5 and 10 min were 7 and 7.

**Fig. 1:**
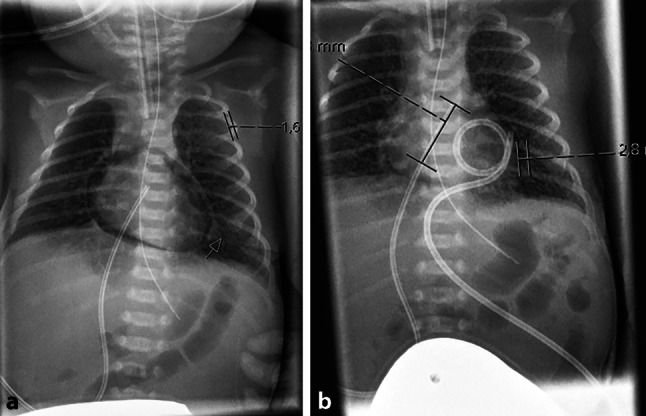
**a** Chest X-ray demonstrating circular pneumopericardium, **b** Chest X-ray after insertion of pericardial tube (pigtail)

**Fig. 2:**
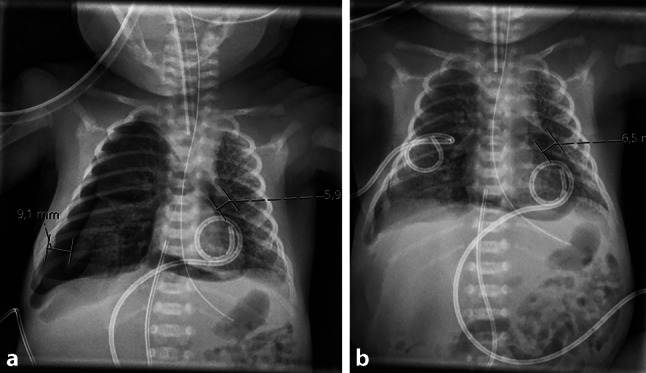
**a** Chest X-ray demonstrating right-sided pneumothorax, **b** Chest X-ray after insertion of a chest tube (pigtail) catheter

The infant was given two doses of surfactant because of severe respiratory distress syndrome and due to worsening respiratory function, conventional mechanical ventilation was switched to high frequency oscillatory ventilation. On day 2 of life, an echocardiography was performed for PDA (Patent ductus arteriosus) assessment, demonstrating circular air entrapment surrounding the infant’s heart (Video 1). On chest X‑ray, suspected pneumopericardium was confirmed (Fig. 1a), and a pericardial tube was inserted with continuous drainage for 3 days (Fig. 1b). The following day the neonate developed right-sided pneumothorax (Fig. 2a), which mandated the insertion of a chest drain (Fig. 2b).

After 3 days, the tubes were removed, and the infant was extubated on day 9 of life. On cerebral ultrasonography, bilateral grade 2 intraventricular hemorrhage was noted. The infant was discharged home without further sequelae at 37 completed weeks of gestation and a body weight of 2785 g.

This report highlights the early and unusual detection of a pneumopericardium by echocardiography prior to potential development of cardiocirculatory compromise. It is important to take pneumopericardium into the differential diagnosis when difficulties arise in the visualization of the heart by conventional echocardiography. Pneumopericardium is associated with a high mortality rate, and may be effectively treated by immediate insertion of a pericardial catheter [1–3].

### Supplementary Information

Echocardiography demonstrating pericardial air entrapment
